# Transcriptional and methylation outcomes of didehydro-cortistatin A use in HIV-1–infected CD4^+^ T cells

**DOI:** 10.26508/lsa.202402653

**Published:** 2024-08-01

**Authors:** Luisa P Mori, Michael J Corley, Andrew T McAuley, Alina Pang, Thomas Venables, Lishomwa C Ndhlovu, Matthew E Pipkin, Susana T Valente

**Affiliations:** 1 The Skaggs Graduate School of Chemical and Biological Sciences, The Scripps Research Institute, Jupiter, FL, USA; 2 Department of Immunology and Microbiology, The Herbert Wertheim UF Scripps Institute for Biomedical Innovation & Technology, Jupiter, FL, USA; 3 Department of Medicine, Division of Infectious Diseases, Weill Cornell Medicine, New York, NY, USA

## Abstract

Ongoing viral transcription from the reservoir of HIV-1 infected long-lived memory CD4^+^ T cells presents a barrier to cure and associates with poorer health outcomes for people living with HIV, including chronic immune activation and inflammation. We previously reported that didehydro-cortistatin A (dCA), an HIV-1 Tat inhibitor, blocks HIV-1 transcription. Here, we examine the impact of dCA on host immune CD4^+^ T-cell transcriptional and epigenetic states. We performed a comprehensive analysis of genome-wide transcriptomic and DNA methylation profiles upon long-term dCA treatment of primary human memory CD4^+^ T cells. dCA prompted specific transcriptional and DNA methylation changes in cell cycle, histone, interferon-response, and T-cell lineage transcription factor genes, through inhibition of both HIV-1 and Mediator kinases. These alterations establish a tolerogenic Treg/Th2 phenotype, reducing viral gene expression and mitigating inflammation in primary CD4^+^ T cells during HIV-1 infection. In addition, dCA suppresses the expression of lineage-defining transcription factors for Th17 and Th1 cells, critical HIV-1 targets, and reservoirs. dCA’s benefits thus extend beyond viral transcription inhibition, modulating the immune cell landscape to limit HIV-1 acquisition and inflammatory environment linked to HIV infection.

## Abstract (precis)

The HIV-1 Tat inhibitor didehydro-cortistatin A not only blocks HIV-1 transcription but also induces specific transcriptional and epigenetic changes in CD4+ T cells, promoting a tolerogenic Treg/Th2 phenotype and reducing inflammatory gene expression.

## Graphical Abstract

**Figure:**
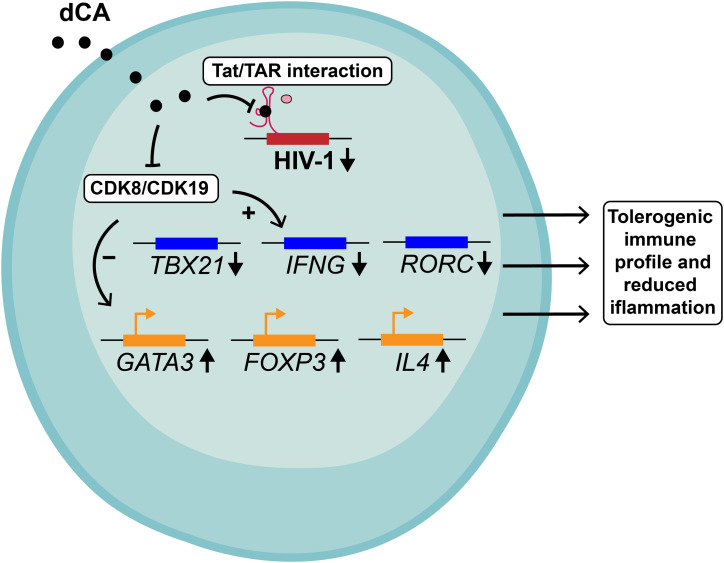

## Introduction

Ongoing viral transcription from integrated HIV persists on daily antiretroviral therapy (ART) and remains a source of complications for people living with HIV (PLWH). Residual plasma viremia and cell-associated (CA)-HIV RNA in blood and tissues are associated with immune activation and are predictive of post-treatment rebound and disease progression (1, 2, 3, 4). The inclusion of specific HIV-1 transcriptional inhibitors to the current ART regimen would likely improve these outcomes and may also limit chronic inflammation stemming from the presence of viral transcripts and proteins derived from viral residual transcription. By blocking RNAPII elongation through the provirus, transcriptional inhibitors may also contribute to epigenetic silencing of the HIV provirus via long-term heterochromatinization of the promoter (5, 6). The block-and-lock approach for a functional HIV cure is based on the premise of epigenetic silencing of the proviral reservoir, which proposes limiting the occurrence of viral rebound, transmission, and disease progression in the absence of any treatment.

Previous work in our research group identified and characterized the small molecule inhibitor of HIV-1 transcription, didehydro-cortistatin A (dCA). Cortistatin A is a steroidal alkaloid isolated from the marine sponge *Corticium simplex* found in Southeast Asia (7). A cortistatin A analog, dCA, with similar bioactivity, was derived to improve the utility of this small molecule (8). The mechanism of action of dCA was characterized in vitro with various cell models of HIV-1 infection and latency, as well as in vivo in the bone marrow, liver, and thymus humanized mouse model of HIV infection (5, 9). dCA was found to inhibit the interaction of the HIV-1 protein Tat with the transactivation response element RNA hairpin (TAR) by binding the basic domain of Tat and blocking Tat-dependent HIV transcriptional amplification in acutely and chronically infected cell line models of HIV infection (EC_50_ = 1 nM, CC_50_ = 20 µM) (9, 10). dCA appears specific for the Tat basic domain, because it does not interfere with the functions of the HIV Rev protein or the cellular protein HEXIM-1, possessing similar basic domains to Tat (10). The only other known high-affinity ligands for cortistatin A are the Mediator kinases CDK8 and its paralog CDK19 (7), but dCA’s HIV-1 inhibitory activity is independent of these kinases, because RNA interference of CDK8 or CDK19 using shRNAs did not result in changes in HIV-1 transcription (10, 11). Consistent with this result, the cycloheptene ring and nitrogen positioning in the isoquinoline of dCA are critical for Tat binding but not for CDK8 inhibition (10). In essence, analogs were derived in which the two activities were isolated and CDK8 inhibitors were inactive against HIV. Conversely, analogs that lack anti-CDK8/CDK19 activity remain to be highly effective Tat inhibitors (10). In sum, dCA specifically binds to the basic domain of Tat, interfering with the Tat-TAR interaction, a mechanism independent of its anti-CDK19/CDK8 activity (12).

Long-term dCA treatment of various cell models of HIV-1, as well as primary CD4^+^ T cells isolated from ART-suppressed PLWH and HIV-1–infected humanized bone marrow, liver, and thymus mice, resulted in a strong block to viral transcription and reactivation, even once the drug was removed, suggesting an epigenetic lock at the HIV promoter. In fact, increased nucleosome stability and decreased histone acetylation levels (associated with active transcription) were observed with dCA treatment at HIV promoters, thereby limiting the recruitment of RNA polymerase II (RNAPII) (5, 6). Here, we sought to understand the effects of long-term dCA treatment on the expression of both viral and host genes and its correlation with DNA methylation levels in primary CD4^+^ T cells. Exploration of these parameters will shed light on the specificity of dCA and the clinical potential of this unique type of antiretroviral drug.

## Results and Discussion

We previously reported an ex vivo study, dubbed the Kessing study, with cells from PLWH to determine the efficacy of dCA added to an ART regiment as compared to ART alone for a period of 5 wk (5) (Table 1). Here, we compared the genome-wide transcriptional landscape determined by RNA-seq of these primary cells with a subsequent study in which primary human CD4^+^ T cells from people not living with HIV were either left uninfected or infected with the laboratory isolate, NL4-3, and treated with ART and dCA for an equal period of 5 wk.

**Table 1.** Table summarizing clinical and demographical characteristics of the Kessing study participants.

Donor	Date of leukapheresis	Age	CD4 absolute (cells/μl)	CD8 absolute (cells/μl)	CD4/CD8 ratio	Viral load (HIV RNA copy/mL)	Nadir CD4	Date of HIV acquisition	Date of ART initiation	ARVs	Gender	Race	Ethnicity
A	40,956	41	1,177	935	1.26	<50	22	2002	<2005		M		
B	40,723	53	1,183	912	1.3	60		1985	<Feb 2008	Reyataz, Epzicom	M	white	Non-Hispanic
C	41,241	60	1,095	736	1.49	<20	385	1990	<August 2006	Atripla	M	white	Non-Hispanic
D	41,291	44	291	491	0.59	<20	316	2002	<Feb 2010	Reyataz, Norvir, Epzicom	M	white	Non-Hispanic
E	41,290	56	715	709	1.01	140	unknown (low 400 s)	1985	<April 2003	Viramune, Truvada	M	white	Non-Hispanic

**Figure 1.**
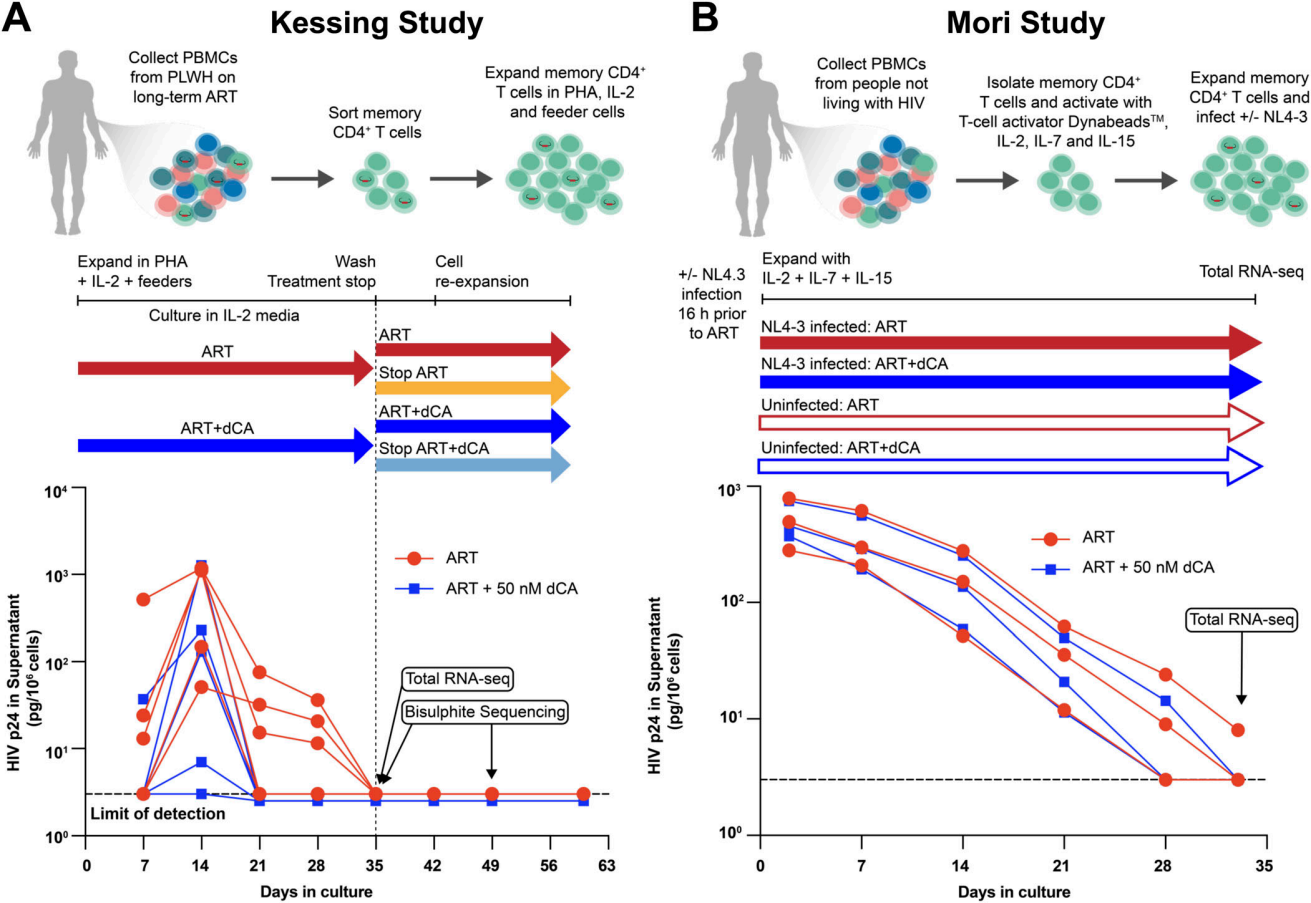
Schematic of treatments of ex vivo human primary CD4+ T cells with didehydro-cortistatin A and ART. **(A)** *Kessing’s study*: CD4^+^ T cells were isolated and expanded ex vivo from virally suppressed people living with HIV. Cells were treated with ART or ART + 50 nM dCA. Viral copies in the supernatant were measured by RT–qPCR per million cells. After all cells were fully virally suppressed, after 35 d of treatment, cells were collected for RNA sequencing and DNA methylation (bisulfite conversion) sequencing. Treatment then continued, or treatment was interrupted for a further 25 d, after which DNAm was measured again by sequencing. Colored arrows represent the different treatment conditions. **(B)** *Mori’s study*: primary CD4^+^ T cells were isolated from people not living with HIV, then either left uninfected or infected with NL4-3, and treated ex vivo with ART or ART + 50 nM dCA. Viral production in the supernatant was measured by p24 ELISA and normalized per million cells. After 33 d of treatment, cells were collected for RNA sequencing.

In the Kessing study, memory CD4^+^ T were purified from PBMCs by FACS and activated ex vivo with PHA, irradiated feeder cells, and interleukin-2 (IL-2). The activation and expansion were performed in the presence of ART to prevent novel rounds of infection and maintain the size of the original viral reservoir of each individual donor. Two treatments were performed in parallel, one with ART+vehicle (DMSO) and the other with ART+50 nM dCA (Fig 1A, circles = ART, squares = ART+dCA). Within 5 wk of treatment, viral suppression in the supernatant was confirmed by RT–qPCR and a fraction of the cells were collected for RNA sequencing and DNA methylation (DNAm bisulfite conversion) sequencing. Either the remaining cells were then treated for another 25 d (Fig 1A, red and dark blue arrows), or treatment was interrupted (“Stop”) until the end of the study (Fig 1A, orange and light blue arrows). On day 49 of treatment, cells were collected from all groups for DNAm sequencing, including those receiving continuous treatment (ART and dCA), or not (treatment stop).

Because the Kessing study included cells from PLWH only, with no uninfected dCA-treated controls, we would be limited in our ability to distinguish HIV inhibition–specific effects on cellular transcriptomics, versus dCA-specific transcriptional changes. To mitigate this, we performed an experiment using memory CD4^+^ T cells from people not living with HIV, treated for the same extent of time and with the same dose of dCA, and infected or not with the laboratory strain NL4-3 (Fig 1B, circles = ART, squares = ART + dCA). This new study was dubbed the Mori study.

**Figure S1.**
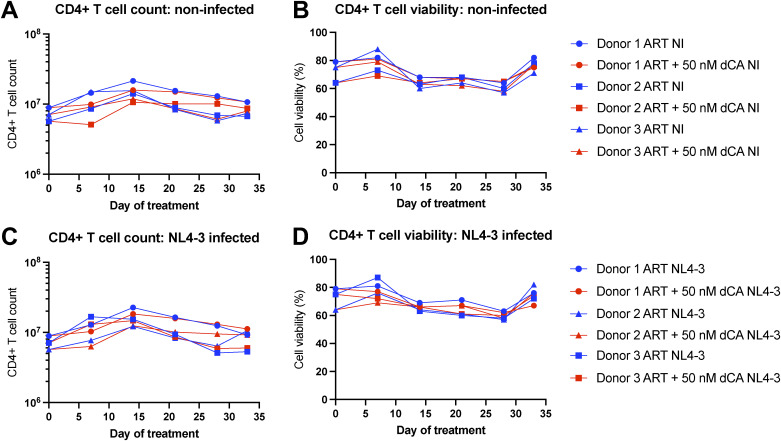
Didehydro-cortistatin A treatment of primary CD4+ T cells does not affect cell viability of growth kinetics. **(A, B)** Cell count and viability of non-infected cells over time measured by trypan blue staining and an automated cell counter. **(B, C, D)** As for (B, C) but for NL4-3–infected cells.

HIV-1 capsid production was monitored over time in the supernatant by p24 ELISA (Fig 1B). Cell growth kinetics and viability were unchanged overtime (Fig S1A–D). In this acute infection model, no significant differences in viral replication were detected with and without dCA, because ART as first-line therapy is already extremely potent and was initiated 16 h post-infection resulting in limited viral replication and a quick and steep decline of viral production to below detection limit in all samples. This experimental setup was planned to replicate as closely as possible the Kessing study. The cell-associated viral RNA was not detected by RT–qPCR in all groups at day 33 post-treatment (Ct > 34), and RNA was sent for sequencing analysis to assess the effects of dCA on cellular gene expression.

### Transcriptional profiling reveals dCA-mediated gene expression changes in primary human CD4^+^ T cells

**Figure 2.**
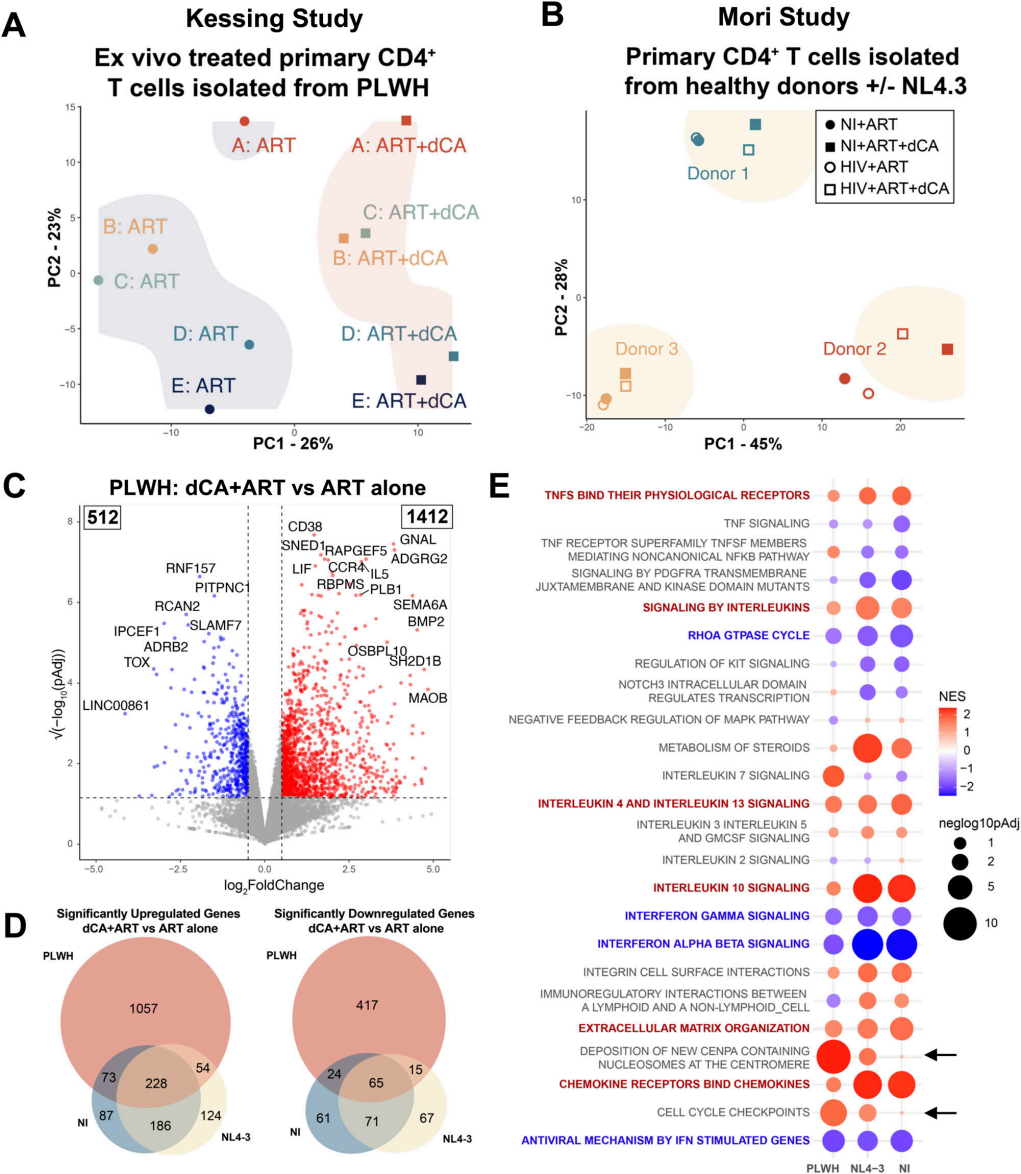
Didehydro-cortistatin A treatment of primary CD4+ T cells leads to both HIV-1–related and HIV-1–unrelated transcriptional changes. **(A)** Principal component analysis of RNA-seq data from CD4^+^ T cells isolated from PLWH and expanded in the presence of ART or ART+dCA for 35 d. **(A, B)** As for (A) but with CD4^+^ T cells isolated from people not living with HIV, either infected or not with HIV-NL4-3, and treated with ART or ART+dCA for 33 d. **(A, C)** Volcano plot of DESeq2 results from PLWH RNA-seq data from (A). Dashed horizontal line: *P*adj = 0.05. Dashed vertical lines: log2FC of 0.5 and −0.5. Blue points: significantly down-regulated genes. Red points: significantly up-regulated genes. **(D)** Proportional Venn diagrams showing the overlap of significantly differentially expressed genes in the three cell models ([log2FC] > 0.5, *P*adj <0.05). **(E)** Bubble plot of a gene set enrichment analysis of the indicated pathways obtained from the Reactome database. In bolded red are pathways enriched in the up-regulated genes in all three cell models. In bolded blue are pathways enriched in the down-regulated genes in all three cell models. NES = normalized enrichment score. Red: genes in this pathway enriched among up-regulated genes in ART+dCA versus ART; and blue: genes in this pathway enriched among down-regulated genes in ART+dCA versus ART.

To determine global effects on cellular gene expression potentially triggered by dCA, RNA-seq analysis was performed integrating the Kessing study (PLWH), as well as the in vitro infection study (HIV) or uninfected (NI) from the Mori study. Principal component analysis of PLWH RNA-seq revealed transcriptional differences between ART- and ART+dCA–treated samples in the first principal component (PC1) (Fig 2A, squares versus circles), as well as differences between donors in PC2 (Fig 2A, different colors). In the HIV and NI samples, the principal component analysis mainly highlighted donor-specific differences (Fig 2B, different colors), with subtler differences from dCA treatment (Fig 2B, squares versus circles) and almost no differences with infection status (Fig 2B, open versus closed shapes). These results are consistent with the undetectable viral levels (Fig 1B). Likely because of limited infection and potent antiretroviral suppression by ART, HIV-1 transcripts could not be reliably detected in any of the HIV-1–infected samples, with mean HIV-1 transcripts per million (TPM) below 0.001 and no significant differences between ART- or ART+dCA–treated cells (data not shown, log2FC = 1, *P*adj = 0.5). Furthermore, HIV-1 transcripts could also not be reliably detected in the Kessing study samples.

**Figure S2.**
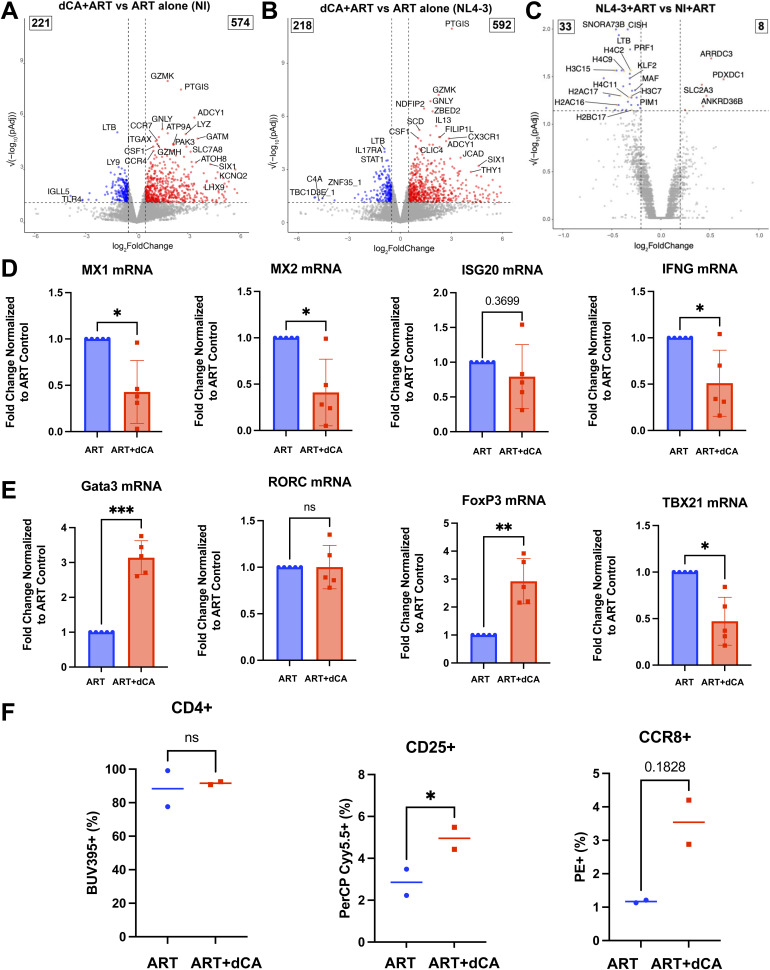
Transcriptional changes in primary CD4+ T cells upon didehydro-cortistatin A treatment. **(A)** Volcano plot of DESeq2 results from RNA-seq data from primary CD4^+^ T cells expanded and treated ex vivo with 50 nM dCA for 5 wk (uninfected = NI). Dashed horizontal line: *P*adj = 0.05. Dashed vertical lines: log2FC of 0.5 and −0.5. Blue points: significantly down-regulated genes. Red points: significantly up-regulated genes. **(A, B)** As for (A) but with cells infected in vitro with the NL4-3 strain of HIV-1. **(C)** Volcano plot of DESeq2 results comparing the RNA expression of primary CD4^+^ T cells expanded, infected with NL4-3, and treated ex vivo with ART versus uninfected ART-treated cells. Dashed horizontal line: *P*adj = 0.05. Dashed vertical lines: log2FC of 0.2 and −0.2. Blue points: significantly down-regulated genes. Red points: significantly up-regulated genes. Orange points: replication-dependent histone genes. **(D)** Expression of IFN-associated genes in PLWH samples down-regulated by dCA treatment, measured by RT–qPCR. **(E)** Expression of lineage-defining transcription factor genes in PLWH samples down-regulated by dCA treatment, measured by RT–qPCR. **(F)** Flow cytometry analysis of the surface expression of CD4 (BUV395), CD25 (PerCP-Cy5.5), and CCR8 (PE) on HIV-negative CD4^+^ T cells cultured with ART or ART+dCA for 5 wk. *P < 0.05, *t* test.

To assess the impact of dCA on gene expression in CD4^+^ T cells from PLWH and people not living with HIV subjected to experimental HIV infection or not (NL4-3 and NI), we conducted a direct comparison of gene expression between cells treated with dCA in conjunction with ART (dCA+ART) and those treated with ART alone. Cells treated with dCA treatment exhibited a greater number of up-regulated genes rather than down-regulated compared to those treated with ART alone (Figs 2C and D and S2A and B, Tables S1, S2, and S3). The intersection of dysregulated genes with dCA treatment in all three cell models (PLWH, NL4-3, and NI) revealed 228 up-regulated and 65 down-regulated genes in common (Fig 2D). Within genes up-regulated by dCA treatment, 87 were unique to NI, and a total of 1,235 were unique to HIV and PLWH samples. Among genes down-regulated by dCA treatment, 61 were unique to NI, and a total of 499 were unique to HIV and PLWH samples. These analyses suggest that dCA treatment leads to the dysregulation of the expression of many genes, some unique to the experimental setting and many common to dCA treatment regardless of the experimental model. In a general manner, it appears that dCA treatment prompts an increase in the expression of more genes than down-regulation, the reason for which is not yet clear. This phenomenon could be related to both the molecular targets of dCA (Tat and Mediator kinases) and some secondary effects.

Table S1. DESeq2 analysis of CD4+ T cells isolated from PLWH treated with 50 nM dCA+ART versus ART alone for 35 d.

Table S2. DESeq2 analysis of CD4+ T cells isolated from healthy donors infected with NL4-3 and treated with 50 nM dCA+ART versus ART alone.

Table S3. DESeq2 analysis of CD4+ T cells isolated from healthy donors treated with 50 nM dCA+ART versus ART alone.

### dCA treatment of HIV-infected CD4^+^ T cells rescues HIV-induced cell cycle and histone gene expression defects, and alters interferon and cytokine signaling pathways in an HIV-independent manner

Next, to identify gene sets or pathways particularly affected by dCA we applied the ReactomePA package in R to the PLWH dataset (13). Because many Reactome gene sets contain overlapping genes, we selected the ones most affected by dCA treatment with non-overlapping genes to perform a gene set enrichment analysis (using the fgsea package in R (14 *Preprint*)) (Fig 2E). The normalized enrichment score indicates whether a certain gene set is enriched in the up-regulated (red) or down-regulated (blue)genes in each dataset.

We found that dCA treatment was associated with the increased expression of replication-associated histone genes (Reactome Pathway: Deposition of new CENPA-containing nucleosomes at the centromere) and cell cycle checkpoint genes (Reactome Pathway: Cell cycle checkpoints), as evidenced by a positive enrichment score, and only noted in the HIV and PLWH samples and not observed in the samples without HIV (NI) (Fig 2E, black arrows). When comparing genes differentially expressed between HIV-infected cells treated with ART (HIV+ART) and those without HIV receiving the same therapy (NI+ART), we found only 41 genes that were differentially expressed (Table S4, Fig S2C). This outcome was somewhat expected because even though HIV-1 replication typically modulates cellular gene expression, at the time of RNA-seq analysis (day 35), HIV-1 replication was close to or at undetectable levels in most samples. Among these 41 differentially expressed genes, eight were replication-dependent core histone genes (*H3C15*, *H4C2*, *H4C9*, *H2AC17*, *H3C7*, *H4C11*, *H2AC16*, and *H2BC17*), all modestly down-regulated (log2FC < −0.20, *P*adj < 0.05; Fig S2C, orange points). The human genome encodes 16 H2A genes, 22 H2B genes, 14 H3 genes, 14 H4 genes, and 6 H1 genes. The expression of these core histone proteins is regulated during the cell cycle, given their requirement for packaging chromatin during DNA replication (15, 16). In fact, over half of the replication-dependent histone genes were down-regulated upon HIV infection (43 of the 72 with a log2FC < 0 and *P* < 0.05; see Table S4). These results suggest that HIV-1 infection may be down-regulating cell cycle histone gene expression; of note, the HIV-1 protein Vpr is known to promote cell cycle arrest via inhibition of p34cdc2/cyclin B and blocking proliferation of CD4^+^ T cells (17, 18, 19, 20). Conversely, the higher expression of these replication-associated histone genes was observed in HIV-infected dCA+ART–treated cells than HIV-infected ART-only–treated cells, but no changes observed in uninfected cells. It is thus plausible that transcriptional inhibition of HIV-1 provided by dCA during the course of the 35-d treatment (5) rescues back to homeostatic levels the expression of histone and cell cycle genes otherwise down-regulated by HIV (Fig 2E, black arrows). Altogether, results suggest that dCA does not directly alter the expression of histone proteins and cell cycle genes. Rather, the dysregulation of cell cycle and histone genes is related to the levels of HIV-1 expression. Future studies should address whether these RNA changes in histone and cell cycle genes translate into changes at the protein level, and whether dCA treatment reverses this phenotype.

Table S4. DESeq2 analysis of CD4+ T cells isolated from healthy donors infected with NL4-3 and ART-treated versus uninfected (NI) ART-treated cells.

We also noted several gene sets altered upon dCA treatment in both infected and uninfected cells. Most notably, we observed enrichment of interferon signaling genes in the genes down-regulated with dCA treatment and enrichment of chemokine and specific interleukin signaling genes among the genes up-regulated by dCA (Fig 2E, genes in bold blue and red, respectively). Interleukin-related pathways appeared to be dominated by an up-regulation of T helper 2 (Th2) signature cytokine genes *IL4*, *IL5*, *IL13*, and *IL3*. *IL10* was also significantly increased with dCA in cells from people not living with HIV (HIV and NI), but not in cells from PLWH. This up-regulation of IL-10–related genes is consistent with the known role of CDK8, inhibited by dCA, in negatively regulating IL-10 in myeloid cells (21). Down-regulation of interferon-induced genes such as *MX1*, *MX2*, *RSAD1*, and *IFIT5* appeared mainly responsible for the enrichment of interferon signaling pathways in down-regulated genes in all three cell models.

In sum, the transcriptional effects of dCA treatment in primary human CD4^+^ T cells were strikingly consistent across different experimental, infection, and T-cell activation conditions. These transcriptional effects include, most notably, changes in cytokine, chemokine, and interferon signaling pathways.

### dCA suppresses interferon gamma–induced gene responses in CD4^+^ T cells, independently of its antiviral activity

**Figure 3.**
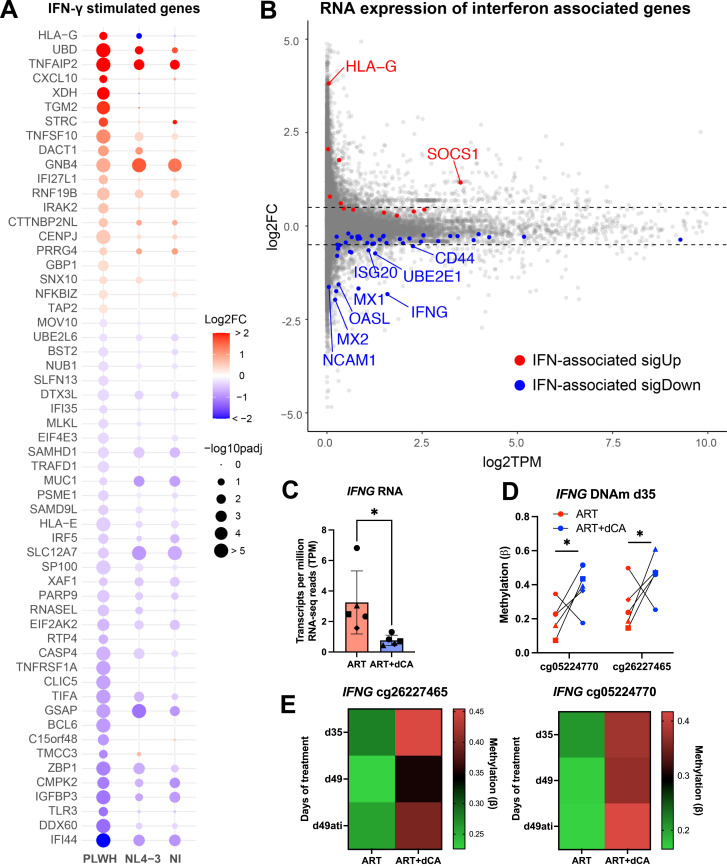
Didehydro-cortistatin A treatment of CD4+ T cells inhibits the expression of interferon-stimulated genes independent of the effects on HIV-1 transcription. **(A)** Bubble plot showing the log2FC in RNA expression (ART+dCA compared with ART alone) of IFN-𝛾–stimulated genes. Statistical significance was represented by the size of each bubble. Genes displayed had a [log2FC] > 0.5 and *P*adj < 0.05 in the PLWH sample. **(B)** MA plot showing the log2FC in gene expression (ART+dCA compared with ART alone) against gene expression levels (transcripts per million, TPM). Genes indicated in red and blue were differentially expressed with *P*adj < 0.05 and [log2FC] > 0. Dashed lines mark a log2FC of −0.5 and 0.5. **(C)** RNA expression of *IFNG* in CD4^+^ T cells from PLWH treated for 35 days with ART alone or ART + dCA. **(D)** DNA methylation (DNAm) of two distinct CpG sites downstream of the TSS of *IFNG* in CD4^+^ T cells from PLWH treated for 35 d with ART alone or ART + dCA. **(E)** DNAm of the two *IFNG* CpG sites after 35 d (d35), 49 d (d49), or 2 wk of treatment interruption after 35 d of treatment (d49ati). Green indicates lower levels of DNAm, and red indicates more DNAm. *P < 0.05. PLWH = CD4^+^ T cells isolated from PLWH. NL4-3 = CD4^+^ T cells isolated from people not living with HIV and infected ex vivo with the NL4-3 strain of HIV. NI = CD4^+^ T cells isolated from people not living with HIV, uninfected.

Next, we assessed the activity of dCA on genes known to be modulated by CDK8 or its paralog CDK19. CDK8/CDK19 bind cyclin C (*CCNC*), Med12, and Med13 forming a kinase module that reversibly associates with the Mediator complex to modulate the expression of a specific set of genes (22, 23, 24). CDK8/CDK19 have also been implicated in promoting the expression of IFN-γ–related genes through RNAPII pause/release (21). As such, we focused on IFN-stimulated gene expression in all three cell samples treated with dCA, including uninfected primary CD4^+^ T cells. We observed significant enrichment of IFN-related gene pathways among down-regulated genes, suggesting a mechanism that is independent of changes in HIV-1 gene expression (Fig 2E). Thus, we extracted a list of genes constituting the three IFN-associated pathways (Reactome Pathways: Antiviral mechanism by IFN-stimulated genes, Interferon gamma signaling, and Interferon alpha/beta signaling) and visualized their differential expression (log2FC) in all three cell models (Fig 3A). We also plotted the log2FC in the PLWH model upon dCA treatment versus their expression levels (log2TPM) (Fig 3B). Of note, most IFN-associated genes were down-regulated with dCA treatment, many by more than 1.4-fold (Fig 3A and B, dotted lines), including *MX1*, *MX2*, *ISG20*, and *IFNG* (Fig 3B, blue points), and results were confirmed by RT–qPCR (Fig S2D). A few IFN-associated genes were up-regulated, such as *HLA-G* and *SOCS1*, upon dCA treatment (Fig 3B, red points). However, *HLA-G* is not usually expressed in T cells and the extremely low levels detected in the dCA-treated samples are unlikely to be of great biological significance (0.05–0.1 TPM). The twofold increase in *SOCS1* expression further supports the observed decrease in IFN-associated gene expression, because SOCS1 primarily acts as a negative feedback inhibitor of the Janus kinase/signal transducer and activator of transcription (JAK-STAT) signaling cascade during cytokine signaling (25). Interferon alpha or beta transcripts (*IFNA* or *IFNB*), primarily produced by macrophages, fibroblasts, endothelial cells, and plasmacytoid dendritic cells, were, as expected, not reliably detected in these CD4^+^ T cells (Tables S1, S2, S3, and S4). There was also no change in the expression of the type I IFN receptors *IFNAR1/2* upon dCA treatment.

Altogether, these results are consistent with Mediator kinase positive regulation of IFN-γ–stimulated gene expression in MEFs through regulation of RNAPII pausing (21).

### dCA impacts epigenetic DNA methylation states of CD4^+^ T cells

**Figure S3.**
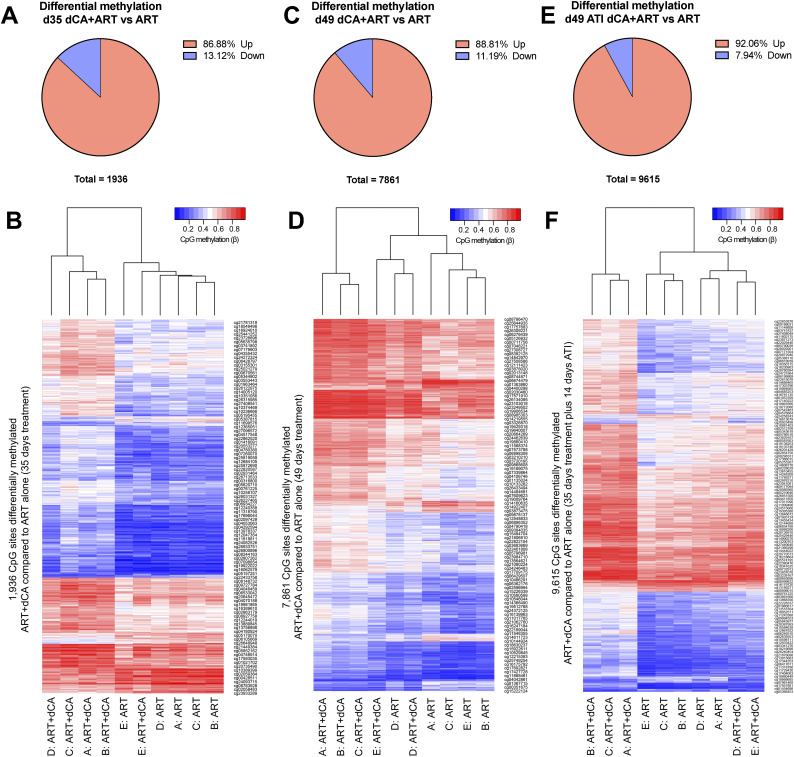
Didehydro-cortistatin A–induced DNA methylation changes increase over time. **(A)** Pie chart displaying the number and proportion of CpG sites that had significantly different DNA methylation levels in the d35 samples (35 d of dCA treatment) (*P*adj <0.05). CpG sites with more methylation with dCA treatment are shown in red and those with less in blue. **(B)** Heatmap displaying each differentially methylated CpG site in the rows, and each PLWH (Donors A–D) sample in the columns. **(A, B, C, D)** As for (A) and (B), but after 49 d of dCA treatment (d49). **(A, B, E, F)** As for (A) and (B), with 35 d dCA treatment but after a 2-wk treatment interruption (d49ati).

In parallel to RNA-sequencing analysis, we profiled longitudinal genome-wide DNA methylation (DNAm) of the Kessing study samples. DNA methylation analysis was performed with samples from day 35 (matching RNA-seq data [PLWH]) and day 49 of dCA treatment (d49), as well as samples with a two-week washout period after treatment interruption referred to as d49ati (Tables S5, S6, and S7). CD4^+^ T cells were found to harbor 1,936 significantly differentially methylated CpGs associated with d35 of dCA treatment, increasing to 7,861 sites by day 49 (Fig S3A–D). Interestingly, these DNAm changes associated with dCA treatment persist and continued to develop even once treatment was interrupted, with 9,615 differentially methylated CpG sites compared with ART alone after the 2-wk washout period (Fig S3E and F). The number of intact proviruses in cells from PLWH was too low to reliably sequence any provirus-associated DNA methylation, even with specialized probes designed for the autologous virus in each donor.

Table S5. DNAm bisulfite–sequencing DESeq2 analysis of CD4+ T cells isolated from PLWH treated with 50 nM dCA+ART versus ART alone for 35 d.

Table S6. DNAm bisulfite–sequencing DESeq2 analysis of CD4+ T cells isolated from PLWH treated with 50 nM dCA+ART versus ART alone for 49 d.

Table S7. DNAm bisulfite–sequencing DESeq2 analysis of CD4+ T cells isolated from PLWH treated with 50 nM dCA+ART versus ART alone for 35 d followed by a 14-d analytical treatment interruption.

In agreement with the RNA-seq data from the d35 samples of the Kessing study (PLWH), where the expression of *IFNG* RNA decreased from around 3 TPM to less than 1 TPM (Fig 3C), we observed two CpG sites close to the promoter of *IFNG*, which were differentially methylated upon dCA treatment (Fig 3D). One differential CpG site (cg26227465) was located between the transcription start site (TSS) and 200 nt upstream of the TSS of the *IFNG* gene, and the other between the TSS and 1,500 nt upstream of the TSS (cg05224770). Increased methylation of these two sites has previously been shown to associate with decreased *IFNG* expression (26). Both sites presented increased DNAm with dCA treatment for 35 d (Fig 3D). We tracked these two CpG sites longitudinally and found these to be consistently more methylated in dCA-treated than ART-alone samples, and methylation persisted even 2 wk post treatment interruption (Fig 3E, d49ati).

A previous study showed that treatment with CA, the parent compound of dCA, of mouse embryonic fibroblasts reduced the expression of interferon-response genes in a CDK8-dependent manner (21). Here, we concur that dCA treatment reduces the expression of many genes related to interferon signaling in primary CD4^+^ T cells. Viral infections are well known for inducing interferon responses, so one might expect an HIV-1 transcriptional inhibitor, such as dCA, to reduce interferon signaling in T cells through reductions in viral load. However, it appears that inhibition is independent of the inhibition of HIV-1 transcription by dCA, because the same effects were observed in uninfected cells (Fig 3A). Rather, it seems likely that the inhibition of IFN-stimulated genes by dCA is a consequence of CDK8 and CDK19 inhibition, as previously published (21).

### dCA treatment of memory CD4^+^ T cells shifts transcriptional profile toward a Th2/Treg phenotype

A notable overlap in gene expression changes was found between the Kessing and the Mori studies, infected or not. Over 200 genes were consistently up-regulated by dCA treatment in PLWH, HIV, or NI samples. These RNA samples were prepared from memory CD4^+^ T cells treated in different ways ex vivo. In the Kessing study, memory CD4^+^ T cells were FACS-isolated from PBMCs from PLWH, activated with PHA, IL-2, and irradiated feeder cells (Fig 1A). On the contrary, in this current study, almost a decade later, memory CD4^+^ T cells were isolated from healthy donor PBMCs by negative selection using antibodies and magnetic beads, and activated using anti-CD3/anti-CD28 stimulation and cultured with IL-2, IL-7, and IL-15. Despite these differences, we observed remarkably similar gene expression signatures (Fig 2D and E). Of particular interest, we noted a significant increase in the expression of specific interleukin genes in all three cell models, namely, *IL4*, *IL5*, *IL13*, and *IL3* (Tables S1, S2, and S3). These cytokines are a signature of T helper 2 (Th2) cell subset, and this observation raised the hypothesis that the balance of CD4^+^ T-cell subtypes might be altered during the in vitro culture with dCA.

**Figure 4.**
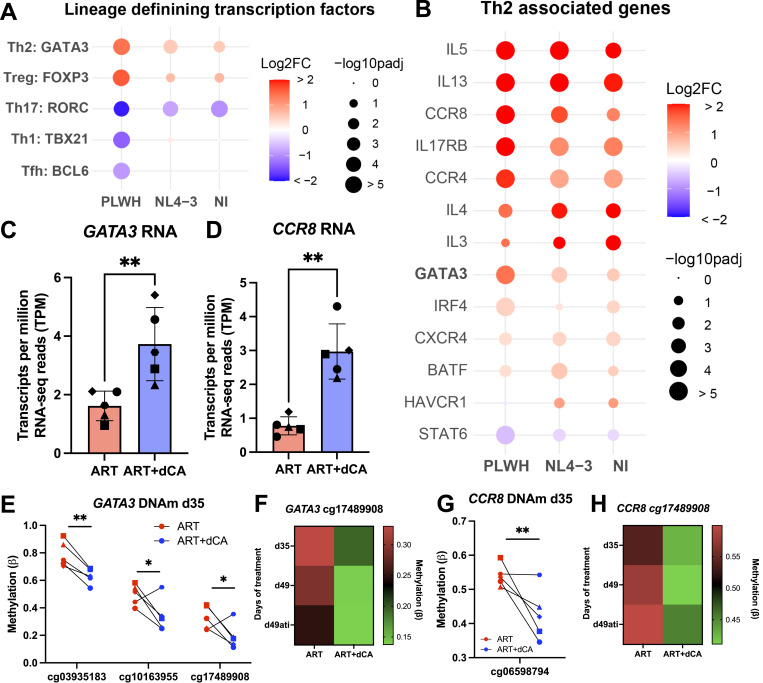
Didehydro-cortistatin A treatment drives the expression of Th2 and Treg signatures in primary CD4^+^ T cells. **(A)** Bubble plot showing the log2FC in RNA expression (ART+dCA compared with ART alone) of indicated CD4^+^ T-cell lineage–defining transcription factor genes. Statistical significance was represented by the size of each bubble. **(B)** Bubble plot showing the log2FC in RNA expression (ART+dCA compared with ART alone) of indicated Th2 signature genes. **(C)** RNA expression of *GATA3* in CD4^+^ T cells from PLWH treated for 35 days with ART alone or ART + dCA. **(D)** RNA expression of *CCR8* in CD4^+^ T cells from PLWH treated for 35 days with ART alone or ART + dCA. **(E)** DNA methylation (DNAm) of three distinct CpG sites downstream of the TSS of *GATA3* in CD4^+^ T cells from PLWH treated for 35 d with ART alone or ART + dCA. **(F)** DNAm of one *GATA3* CpG site after 35 d (d35), 49 d (d49), or two weeks of treatment interruption after 35 d of treatment (d49ati). Green indicates lower levels of DNAm, and red indicates more DNAm. **(G)** DNA methylation (DNAm) of a CpG site downstream of the TSS of *CCR8* in CD4^+^ T cells from PLWH treated for 35 d with ART alone or ART + dCA. **(H)** DNAm of the *CCR8* CpG site after 35 d (d35), 49 d (d49), or two weeks of treatment interruption after 35 d of treatment (d49ati). *P < 0.05, **P < 0.01. PLWH = CD4^+^ T cells isolated from PLWH. NL4-3 = CD4^+^ T cells isolated from people not living with HIV and infected ex vivo with the NL4-3 strain of HIV. NI = CD4^+^ T cells isolated from people not living with HIV, uninfected.

To examine the expression of CD4^+^ T-cell lineage–defining transcription factors T-bet (*TBX21*, T helper 1, Th1), GATA3 (*GATA3*, Th2), RORγ (*RORC*, T helper 17, Th17), FOXP3 (*FOXP3*, regulatory T, Treg), and BCL6 (*BCL6*, follicular T, Tfh), we compared their RNA expression in cells treated with ART+dCA to ART only (Fig 4A). Notably, we found the expression of *GATA3* and *FOXP3*, the lineage-defining transcription factors for Th2 and Treg cells, respectively, was increased in all three sample conditions upon dCA treatment (Fig 4A). Concurrently, a decrease in the Th17 cell lineage–defining transcription factor *RORC* was detected in all three sample conditions, along with a significant decrease in Th1 and Tfh lineage–defining transcription factors, *TBX21* and *BCL6*, respectively, in PLWH cells treated with dCA, but not HIV and NI cells treated with dCA (Fig 4A). We confirmed these changes in the expression of *GATA3*, *FOXP3*, and *TBX21* by RT–qPCR (Fig S2E). In addition, with dCA treatment we observed significant increases in the expression of Th2 signature genes including transcription factors *IRF4* and *BATF*; cytokines *IL5*, *IL13*, *IL4*, and *IL3*; and Th2 cell surface markers *CCR8*, *CCR4*, *CXCR4*, and *IL17RB* (Fig 4B). The increase in *GATA3* and *CCR8* expression correlated with loss of methylation of these genes (Fig 4C–H). *GATA3* has three CpG sites within the gene body that were differentially methylated upon 35 d of dCA treatment. The methylation loss at these three sites persisted over time, even when treatment was interrupted (Fig 4E and F), suggesting CD4^+^ T-cell phenotype changes are well established and persist beyond dCA treatment. A CpG site sits just downstream of the TSS of the *CCR8* gene, which displayed losses in methylation after 35 d of dCA treatment compared with ART alone, and again, these differences persist over time (Fig 4D, G, and H). To determine whether these transcriptional changes resulted in changes at the protein level, we measured the surface expression of CCR8 and CD25, markers of Th2 and Treg cells, respectively (Fig S2F), in an independent experiment where CD4^+^ T cells from two HIV-negative donors were treated for 5 wk with ART or ART+dCA. In agreement with the RNA-seq analysis, the expression of CD25 and CCR8 was increased in dCA-treated cells, whereas the proportion of CD4^+^ cells was unaffected.

Taken together, these results suggest that dCA treatment of primary memory CD4^+^ T cells promotes a Th2/Treg phenotype in culture, likely via inhibition of Mediator kinases CDK8 and CDK19 because the same phenotype is observed in uninfected CD4^+^ T cells and thus is likely independent of the inhibition of Tat by dCA. Our results corroborate a recent study, which showed that treatment of primary naïve human and mouse CD4^+^ T cells treated with dCA differentiated into Th2 and Treg, and blocked differentiation of Th1 and Th17 cells (27). The authors show this phenotype is largely dependent on CDK8 inhibition by dCA, and seemingly independent of previously known roles of STAT1/STAT3 phosphorylation by CDK8. They propose that dCA drives this Th2, Treg phenotype via a previously undefined CDK8-GATA3-FOXP3 pathway (27). In our study, the isolated memory CD4^+^ T cells have already undergone differentiation; however, ∼threefold increases in both *GATA3* and *FOXP3* expression were detected, with a concurrent 50–75% reduction in T-bet (*TBX21*), RORγ (*RORC*), and *BCL6* expression (Tables S1, S2, and S3). It remains unclear precisely how dCA triggers *GATA3* expression and whether dCA is driving an induction of previously silenced *GATA3* and *FOXP3* loci or instead it is favoring the survival of Th2 and Treg cells over Th1 and Th17 cells, and these questions warrant further exploration.

Understanding the effects of dCA treatment on CD4^+^ T-cell transcriptional and epigenetic phenotypes is vital as we move forward in the clinical pipeline with HIV-1 transcriptional inhibitors. We need to parse out the effects of HIV-1 inhibition itself on cellular gene expression profiles, from the transcriptional effects of the inhibitors themselves, and here, we made strides forward in this regard. We found that HIV-1 inhibition by dCA reverses some transcriptional defects that occur with HIV-1 infection, including HIV-1–induced reduction in the expression of cell cycle and histone genes. Furthermore, dCA reduces interferon-stimulated gene expression; thus, the addition of dCA to current ART holds promise for reducing the chronic inflammation that is observed during long-term HIV-1 infections, even when ART was suppressed. Finally, we found that dCA treatment of fully differentiated memory CD4^+^ T cells skewed the population to a more Th2/Treg phenotype, which is associated with a more tolerogenic profile in tissues (28). Importantly, higher levels of immune exhaustion and immune activation have been associated with reduced Treg cell numbers in viremic chronically HIV-1–infected patients, which were partially rescued by virologic control after ART (29). In addition, the expression of *FOXP3* in primary human CD4^+^ T cells has been shown to significantly inhibit HIV-1 infection via inhibition of NFAT activity at the HIV-1 promoter, which may reduce the permissiveness of this T-cell subset to HIV-1 infection (30).

With respect to the increased expression of Th2 signature genes, Th2 cells have been shown to contain lower levels of HIV-1 DNA in PLWH on long-term ART, and CD4^+^ T cells enriched for Th1/17 polarization were more susceptible to HIV-1 infection in ex vivo assays (31). Th17 cells are also the preferential target for HIV-1 infection in the gut, and because of their longevity and proliferative nature, they serve as ideal reservoirs for HIV-1 DNA (32). In fact, Th1/17 CD4 T cells are the major long-term persistent reservoir of HIV-1 DNA in patients receiving ART (31). Thus, tipping the balance of CD4^+^ T-cell subsets from the Th1/Th17 axis toward the Treg/Th2 axis with dCA treatment may be an added benefit to the HIV-1 transcriptional inhibition it already provides. dCA may not only reduce ongoing HIV-1 transcription during ART that is associated with chronic immune activation, but also, through inhibition of CDK8/CDK19, reduce interferon responses by lowering chronic inflammation in tissues of PLWH, increase the number of Treg cells further limiting inflammation, and finally reduce the number of cells that serve as the major reservoir for HIV-1 infection. It is possible, however, that altering the cytokine and T-cell subtype profiles in tissues may unpredictably modulate HIV-1 infection. Thus, it is critical that all these parameters be assessed in non-human primate models of HIV-1 infection and determine whether analogs of dCA lacking CDK8 activity need to be further developed.

### Limitations of the study

We examined the transcriptional effects of the small molecule dCA in memory CD4^+^ T cells from two studies (the Kessing and Mori studies) conducted several years apart with different T-cell activation protocols. We focused on genes and pathways that were common to all dCA-treated samples; thus, we may have failed to detect the effects of dCA only observable with certain activation and/or expansion conditions. Despite these differences, we identified an up-regulation of Th2 and Treg signature genes and a down-regulation of Th1 and Th17 signature genes upon dCA treatment, corroborating previous studies, particularly that of Arnett et al (2021). Here, we have not confirmed that these transcriptional changes translated in changes at the protein level, and future work will assess whether dCA is functionally preprogramming memory CD4^+^ T cells away from Th1/Th17 and toward Th2/Treg subtypes or simply enhancing the survival of Th2 and Treg cells.

## Materials and Methods

### Isolation, activation, and culture of primary CD4^+^ T cells from people not living with HIV

For the Kessing study, refer to Kessing et al (2017). For the Mori study, human PBMCs from three people not living with HIV were obtained from buffy coats (LifeSouth Community Blood Centers) by density centrifugation as previously described (33). Primary memory CD4^+^ T cells were isolated by negative selection (#19157; STEMCELL Technologies) and activated with a 1:2 ratio with Dynabeads Human T-Activator (#11131D; Gibco) in the presence of 6 ng/ml IL-2 (MACS, #130-097-748; Miltenyi Biotec), 36 ng/ml IL-7 (#247ILB005CF; R&D Systems), and 36 ng/ml IL-15 (#207IL005CF; R&D Systems). The cells were cultured in RPMI 1640 media (#11875-119; Gibco) supplemented with 1xMEM vitamin solution (#11120052; Life Technologies), 1xNEAA (#11140076; Life Technologies), 1xNa pyruvate (#11360070; Life Technologies), 1xPen/Strep/L-glut (#10378016; Life Technologies), 0.01 M Hepes, pH 8.0 (#15630130; Life Technologies), 1xL-Asp/L-Arg/folic acid mix (house-made), and 10% FBS (catalog no. FS-0500-AD; Atlas Biologicals).

### Infection and drug treatment of memory CD4^+^ T cells

3 d post-activation, cells were infected with 5 ng p24 NL4-3 per 10^6^ cells at a cell density of 2 million/ml; note that half of the donor cells were left uninfected for control. Virus was simply added to the media, and cells were mixed every 20 min for 2 h and then left overnight. Cells were then pelleted by centrifugation after 12 h and resuspended in fresh T-cell media (uninfected controls were treated in the same way) at a concentration of 1 million/ml. Cells were then divided into ART+dCA or ART-alone treatments (including the uninfected controls). Cells were then maintained in the following ART cocktail: 200 nM lamivudine (#8146; HIV Reagent Program), 200 nM raltegravir (MK-0518; Selleck Chemicals), and 100 nM efavirenz (#0977; HIV Reagent Program). Didehydro-cortistatin A (dCA) was synthesized by Dr. Ravi Natarajan, Thimble Therapeutics, and was used at a concentration of 50 nM. Half-medium changes were performed every 3–4 d as needed, with a fresh drug being added to maintain the concentration. Every 7 d, the supernatant was collected for p24 ELISA, and cells were counted and reseeded at a concentration of 1.5 million/ml. After 5 wk of treatment (33 d), cells were collected for RNA-sequencing analysis.

### p24 ELISA

The amount of the HIV p24 capsid protein produced in the supernatant of cultured cells was quantified using the antigen capture assay kit from Advanced BioScience Laboratories, Inc. (no. 5447) and performed according to the manufacturer’s protocol.

### RNA extraction, DNase treatment, and RT–qPCR

Total RNA was extracted from cells using an RNeasy kit (no. 74106; QIAGEN). RNA was DNase-treated using the TURBO DNA-free kit (no. AM1907; Invitrogen). cDNA was synthesized using random hexamer primers and SuperScript III First-Strand Synthesis Kit (no. 18080051; Invitrogen). RT-qPCR was performed using SensiFAST SYBR No-ROX Kit (#BIO-98020; Bioline). Samples were run in triplicate. Target gene mRNA expression was normalized to RPL13A mRNA expression (primer purchased from Bio-Rad: PrimePCR SYBR Green Assay RPL13A human no. 10025637), and the relative abundance was calculated (ΔΔCt method). The following primers were used:

MX1_mRNA_F—GCAATCAGCCTGCTGACATT.

MX1_mRNA_R—CCAGATCAGGCTTCGTCAAGA.

MX2_mRNA_F—GGACAGGGGCACTGAGAAAA.

MX2_mRNA_R—CAACGGGAGCGATTTTTGGA.

ISG20_mRNA_F—GAGTGAGCGCCTCCTACACAA.

ISG20_mRNA_R—GGCTCGGATTCTCTGGGAGA.

IFNG_mRNA_F—GCTGTTACTGCCAGGACCC.

IFNG_mRNA_R—TTTTCTGTCACTCTCCTCTTTCC.

RORC_mRNA_F—AGCCTCACGGGAGCTG.

RORC_mRNA_R—CGTAGTGGATCCCAGACGAC.

TBX21_mRNA_F—AGTGGGTGCAGTGTGGAAAG.

TBX21_mRNA_R—CTGGAGCACAATCATCTGGGT.

FoxP3_mRNA_F—ACACTGCCCCTAGTCATGGT.

FoxP3_mRNA_R—ATCCACCGTTGAGAGCTGGT.

GATA3_mRNA_F—AGGGCCACGGTGCAGAGGTA.

GATA3_mRNA_R—GTGGTGGCTGCCCAGGGCTT.

### Total RNA sequencing

CA-RNA was extracted as described above. Total RNA was quantified using Qubit 2.0 Fluorometer (Invitrogen) and evaluated on Agilent 2100 Bioanalyzer Nano Chip for quality assessment. The DNase-treated total RNA (350 ng input) was depleted of ribosomal RNA using probes provided by the NEBNext rRNA depletion module (Cat. #: E6310L; NEB) according to the manufacturer’s recommendations. The library preparation from the rRNA-depleted RNA was conducted according to NEBNext Ultra II Directional RNA Kit (Cat. #: E7760; NEB) guidelines. Briefly, the samples were chemically fragmented in a buffer containing divalent cations by heating to 94°C for 15 min. The fragmented RNA was random hexamer–primed and reverse-transcribed to generate the first-strand cDNA. The second strand was synthesized after removing RNA template and incorporating dUTP in place of dTTP. The double-stranded cDNA was then end-repaired and adenylated at their 3′ ends. A corresponding T nucleotide on the adaptors was used for ligating the adaptor sequences to cDNA. The adaptor loop contains a dUTP that is removed along with all other incorporated Us in the second strand by treatment with USER enzyme (Uracil-Specific Excision Reagent). The degradation of the second strand in this step preserves directional sequencing of the intact first strand, thus preserving strand information of the RNA. The adaptor-ligated DNA was purified using magnetic beads and PCR-amplified using nine cycles to incorporate a unique barcode and generate the final libraries. The final libraries were purified using magnetic beads to remove any remaining primers and adaptors, validated on a TapeStation D1000 tape, normalized to 4 nM, and pooled equally. The final pool was loaded onto the NextSeq 500 (PLWH samples) or NextSeq 2000 (NL4-3 and NI samples) P3 flow cell (Illumina, San Diego, CA) at 750 pM final concentration and sequenced using 2 × 50 bp paired-end chemistry. On average, we generated 39 to 53 million reads passing the filter per sample.

### Total RNA-sequencing analysis

The read quality was assessed using FastQC (Braham Bioinformatics) and MultiQC (34). Raw FASTQC files were filtered for read quality using trim-galore version 0.6.1 with the following options: trim_galore ‐‐paired ‐‐length 24 ‐‐stringency 3 (https://www.bioinformatics.babraham.ac.uk/projects/trim_galore/). A custom genome was created by merging the human genome (GRCh38.p14) and the HIV-1 genome (NL4-3: GenBank: AF324493.2, or PLWH autologous virus—a consensus sequence was constructed from the DNA methylation sequencing). To align transcripts, salmon version 0.14.1 was used with the ‐‐validateMappings option (35). Aligned transcriptomes were mapped to genes, and differential gene expression was called using tximport version 1.28.1 and DESeq2 version 1.40.2, respectively (36, 37). Raw counts were imported using the DESeqDataSetFromTximport function from DESeq2, and differential expression analysis was performed using DESeq2. Variation in the data was accounted for in the design formula using “∼donor + treatment” for the Kessing study samples and “∼infection + donor + treatment” for the Mori study samples. TPMs were determined by normalizing counts to gene length (in kb) and then to library size. All plots were made in R using ggplot2 (38). ReactomePA was used to conduct gene pathway analysis (13, 39). The fgsea package was used to conduct gene set enrichment analysis with publicly available gene sets (14 *Preprint*, 40, 41, 42).

### Flow cytometry

CD4^+^ T cells were washed with PBS and then stained with a viability dye (1:5,000 dilution of eBioscience Fixable Viability Dye eFluor 780, #65-0865-14) at 4°C for 20 min. Cells were then washed with staining buffer (2% FBS in PBS) and then resuspended in staining buffer plus CD4-BUV395 (#563552; BD), CCR8-PE (#50-237-6930; BioLegend), and CD25-PerCP-Cy5.5 antibody (#560503; BD) for 15 min at 4°C (protected from light). Cells were then washed with staining buffer and fixed with 1% formaldehyde for 10 min at room temperature. Cells were then washed and resuspended in staining buffer and stored in the dark until acquired on a BD Symphony flow cytometer. Cells were gated on live cells, singlets, and CD4 expression. Then, the percentage of CD4^+^ cells expressing CCR8 or CD25 was measured.

### Quantification of DNA methylation

Three hundred nanograms of DNA per sample was bisulfite-converted using the EZ DNA Methylation kit (#1154D88; Zymo Research) according to the manufacturer’s protocol. The bisulfite-converted DNA samples were randomly assigned to a chip well on Infinium HumanMethylationEPIC v1.0 BeadChip (43), amplified, hybridized onto the array, stained, washed, and imaged with the Illumina iScan SQ instrument to obtain raw image intensities. Raw Methylation EPIC array IDAT intensity data were loaded, preprocessed, filtered, and normalized in the R statistical programming language (http://www.r-project.org) using the Sesame R package (44). Differentially methylated sites for comparisons between control and dCA groups were calculated using the limma package linear model (45, 46).

### Statistical analysis

Graphs and analysis were performed using GraphPad Prism (version 10.0). Data are presented as the mean ± SEM. *P* < 0.05 was considered statistically significant. Paired two-sided Mann–Whitney *U* tests were performed to determine the statistical significance of the differences between the ART+dCA-treated and the ART control group.

## Supplementary Material

Reviewer comments

## Data Availability

RNA-seq and DNA methylation (bisulfite conversion)–sequencing data generated from the Kessing and Mori studies have been deposited to Gene Expression Omnibus (GEO) and can be found under the accession number GSE272666.
